# Perceptions and Beliefs About Obesity and Bariatric and Metabolic Surgery Among Black and White Men

**DOI:** 10.1007/s11695-025-07878-6

**Published:** 2025-04-23

**Authors:** Ajay A. Myneni, Brooks C. Harmon, Joseph D. Boccardo, Iman Simmonds, Dennis E. N. Daniels, Heather Orom, Ranjit Singh, Gregory G. Homish, Aaron B. Hoffman, Katia Noyes

**Affiliations:** 1 University at Buffalo, State University of New York, Buffalo, USA; 2 Methodist Dallas Medical Center, Dallas, USA

**Keywords:** Black men, Non-Hispanic White men, Obesity, Metabolic and bariatric surgery, Obesity surgery

## Abstract

**Background:**

Although metabolic and bariatric surgery (MBS) is a safe and effective procedure to reduce severe obesity and is covered by most health insurance plans, utilization of MBS is significantly lower among men compared to women. This study identifies unique factors that explain men’s attitude towards MBS.

**Methods:**

The study survey (paper/online) included 129 Black and White men with severe obesity from metropolitan communities in Western New York. Bivariate and multivariate analyses were used to evaluate participants’ personal and community factors influencing their consideration of MBS.

**Results:**

Men willing to undergo MBS had lower education (38% vs. 21% ≤ high school, *p* < 0.05), were less likely to be satisfied with their body weight (27% vs. 48%, *p* < 0.05), more likely to have a physician supporting their weight loss efforts (55% vs. 32%, *p* = 0.03) and discussing MBS treatment (39% vs. 19%, *p* = 0.02), believed that community role models who underwent MBS “lost weight and looked great” (66% vs. 40%, *p* = 0.02) and that MBS was safe and effective (40% vs. 13%, *p* < 0.01), compared to men unwilling to undergo MBS. When adjusted for education level, dissatisfaction with body size (odds ratio, OR = 4.56, 95% confidence interval, CI: 1.16, 18.01) and physician support (OR = 3.71, 95% CI: 1.17, 11.78) remained significantly associated with men’s willingness to undergo MBS. Race and BMI were not associated with willingness to undergo MBS.

**Conclusions:**

Positive attitude toward MBS among men is influenced by self-perception of excess weight, strong physician support and community role models. Improving patient-provider communication about MBS and awareness from community role models may improve MBS utilization among men.

**Supplementary Information:**

The online version contains supplementary material available at 10.1007/s11695-025-07878-6.

## Introduction

Metabolic and bariatric surgery (MBS) is a safe and effective treatment option for patients not responding to non-surgical treatments [1–3]. Despite its effectiveness, utilization of MBS among eligible patients with severe obesity (BMI ≥ 40), and for individuals with BMI 30–40 and obesity-related complications remains below 1% [4–6], with the lowest MBS utilization rates reported among Black men [7, 8].

African Americans overall have 40% lower odds [9] than Whites to utilize MBS [10] with Black men being seven times less likely to undergo MBS than Black women [10, 23]. Lower utilization of healthcare among minority groups is a common phenomenon associated with greater patient financial hardships, access to care and lower health literacy among minority populations [11–13]. Notably, lower utilization of MBS was also observed among socially-disadvantaged population in Canada, despite its public healthcare system [11]. However, the lower rate of MBS use among men compared to women [7, 8, 14, 15] is in contrast with previous evidence demonstrating that women are usually less likely to seek elective surgeries [16, 17], a trend attributed to women’s preferences and perceptions. These include women’s reluctance to disrupt their roles as caregivers, fear of surgery, hesitation to fully disclose the severity of their symptoms, and a preference for greater involvement in treatment decisions. Hence, it is conceivable that the gender differences in undergoing MBS may be influenced by societal stereotypes and weight discrimination, with women facing more social pressure to manage obesity than men [18].

A previous pilot qualitative study conducted among Black men explored intrinsic factors associated with their low utilization of MBS and showed that Black men did not perceive obesity as a serious condition and considered MBS and extreme and dangerous option for obesity management. Further, Black men identified trust in and respectful communication with their physician as very important in making treatment decisions [19]. Building on the principles of the Anderson model of healthcare utilization [20] and previous findings, the current study aims to further examine individual behavioral factors, health beliefs, perceptions and attitudes that may be driving low MBS utilization among men.

## Methods

### Study Population

Adult, English speaking, White and Black men were recruited in community settings Western New York region. Black men were oversampled to achieve an adequate sample size. Eligibility criteria included having a body mass index (BMI) of ≥ 40 or BMI of 35–39 (calculated using participants’ self-reported height and weight) with at least one self-reported obesity-related comorbidity including type 2 diabetes, hypertension, kidney disease, sleep apnea, heart disease, arthritis, liver disease or gastroesophageal reflux disease. Men who previously underwent MBS were not eligible to participate. Those who were undergoing assessments for MBS were not excluded. However, none of the participants who completed the survey met this criteria. The study protocol and materials were reviewed and approved by xxxx’s (omitted for blind review) institutional review board.

### Participant Recruitment

Participants were recruited between March 2021 to September 2022, with flyers displayed at high-traffic locations (e.g., community centers, churches, public libraries, grocery stores, and bus stops) and by study personnel at popular social and sports events. Additionally, to augment recruitment of eligible Black men, the above locations were targeted in communities and zip codes with majority Black population. Potential participants were screened for eligibility by phone/email or on site and informed consent was obtained from eligible men before participation.

### Survey Questionnaire

The study questionnaire was developed based on a previous qualitative study exploring the barriers to utilization of MBS among Black men in the same geographic location as the current study. Responses to the survey questions were framed in the form of yes/no, Likert-type scale and by asking to select single or multiple pertinent answers. More details and the complete instrument are available in Supplemental Table 1. Participant responses to Likert-type scale answers were grouped into meaningful categories as is commonly practiced in testing survey responses [21]. The questionnaire included questions assessing men’s perception of personal health and obesity, weight loss efforts, relationship with physician, and knowledge and attitudes towards MBS. For participants who did not know about MBS before, study staff briefly explained about the safety profile and potential benefits of MBS and encouraged them to consult their physician to learn more and discuss options for weight management if they wish to do so. Participant’s demographics including age, education level, employment status and marital status as well as smoking status (ever/never) were also assessed. Participants completed the survey in-person using paper forms or remotely using Google Forms on their mobile devices. It took 15–30 min to complete the survey, and participants received $20 gift cards as compensation for their time and participation.

**Table 1:** Characteristics of the survey participants by their willingness to consider metabolic and bariatric surgery

Variable	Total n (%)	MBS		
		Unwilling n (%)	Willing n (%)	*p*^*1*^
Sample	129	71	58	
Age, mean (sd)	47.03 (12.84)	48.11 (13.65)	45.76 (11.82)	0.36
Race				
Black	63 (48.8)	30 (42.3)	33 (56.9)	0.10
White	66 (51.2)	41 (57.7)	25 (43.1)	
Body mass index, mean (sd)	42.78 (5.41)	42.28 (5.34)	43.39 (5.48)	0.25
Education (n = 122)				** < 0.05**
High school or less	35 (28.7)	14 (21.2)	21 (37.5)	
Some college or higher	87 (71.3)	52 (78.8)	35 (62.5)	
Currently employed (n = 124)	98 (79.0)	52 (76.5)	46 (82.1)	0.75
Currently married/living with a partner (n = 123)	67 (54.5)	40 (59.7)	27 (48.2)	0.44
Ever smoker (n = 123)	58 (47.2)	30 (44.1)	28 (50.9)	0.45

^1^ from Chi-square or Fisher’s Exact (where Chi tests may not have been valid)

Abbreviations: MBS: metabolic and bariatric surgery

### Outcome of Interest

Willingness of the participants to undergo MBS was assessed as the primary outcome by participants’ “yes” or “no” response to the survey question “Would you consider metabolic and bariatric surgery for management of obesity and obesity complications”. Factors that contributed towards patient’s consideration of surgical treatments for weight loss were also examined.

### Data Analysis

Participant responses from both in-person and mobile surveys were recorded using Google Sheets and imported into SAS 9.4 (Cary, North Carolina) statistical software for analysis. Participant characteristics were examined in the total sample and by the willingness to undergo MBS (Table 1). Participants’ responses to survey questions as a function of willingness to consider MBS were assessed in an unadjusted bivariate analysis (Table 2). Independent sample t-tests for continuous variables (age and BMI) and Chi-square and Fisher’s Exact tests for categorical variables were used to test differences among the study groups. Multiple logistic regression was used to test associations with willingness to consider MBS after adjusting participants’ education level. To confirm that the study had sufficient statistical power, study data was re-analyzed using bootstrapping resampling (n = 1,000) method [22, 23]. The results were not substantively different from the original analysis and were not reported. All statistical tests were two-sided, and alpha was set at 0.05.

**Table 2:** Participants’ perceptions, opinions and beliefs on obesity and metabolic and bariatric surgery by their willingness to consider metabolic and bariatric surgery

Participant opinions, perceptions, and beliefs	Total n (%)	MBS		*P*^1^
		Unwilling n (%)	Willing n (%)	
Sample size, n	129	71	58	
**Perception of personal health and obesity**				
Consider personal health among life’s top priorities^2^	52 (40.9)	30 (43.5)	22 (37.9)	0.53
Neutral/satisfied with own body size^3^	49 (38.6)	34 (47.9)	15 (26.7)	** < 0.05**
Consider themselves to be persons with excess weight or obesity	109 (85.8)	61 (87.1)	48 (84.2)	0.64
Maintaining healthy body weight is important or very important^4^	82 (67.2)	45 (67.1)	37 (67.3)	0.93
**Weight loss efforts**				
Changed both diet and physical activity habits to lose weight	63 (49.3)	36 (51.4)	27 (46.6)	0.07
Family and friends are very supportive in weight loss efforts^5^	67 (53.2)	34 (48.6)	33 (58.9)	0.19
**Relationship with physician**				
Have a PCP for routine medical care	88 (69.9)	45 (66.2)	43 (74.1)	0.07
PCP spoke respectfully when discussing weight	71 (83.5)	35 (79.6)	36 (87.8)	0.57
PCP was not judgmental when discussing weight	59 (70.2)	30 (78.6)	29 (69.1)	0.59
Visited a physician at least once in the past year	115 (89.2)	62 (87.3)	53 (91.4)	0.07
Healthcare provider(s) did not discuss weight/weight loss	12 (9.7)	6 (8.6)	6 (11.1)	0.71
Usual provider or PCP did not give advice or a detailed plan to lose weight or maintain healthy weight	27 (16.0)	17 (18.7)	10 (13.0)	0.60
Doctor was helpful or very helpful in weight loss efforts^6^	54 (42.5)	23 (32.4)	31 (55.4)	**0.03**
**Knowledge and attitude towards MBS**				
Had prior knowledge about MBS	120 (93.7)	68 (95.8)	52 (91.2)	0.53
Knowledge about MBS was from a medical provider	33 (27.5)	13 (19.1)	20 (38.5)	**0.02**
Knew someone personally, who underwent MBS: Believed that this person’s quality of life improved after the surgery.^7^ Believed that this person lost weight and looked great after the surgery	93 (73.8) 51 (54.8) 44 (51.2)	54 (78.3) 21 (48.8) 19 (39.6)	39 (68.4) 30 (60.0) 25 (65.8)	0.21 0.17 **0.02**
Believed that MBS surgery is a safe and effective way to lose weight^7^	31 (24.6)	9 (12.7)	22 (40.0)	** < 0.01**

Abbreviations: MBS: metabolic and bariatric surgery, PCP: primary care physician

^1^ from Chi-square or Fisher’s Exact (where Chi tests may not have been valid); ^2^ a response of 8–10 from a scale of 1–10; ^3^ response from a 5-point scale ranging from very dissatisfied to very satisfied; ^4^ response from a 5-point scale ranging from not important to very important; ^5^ response from a 5-point scale ranging from no role/make it harder to very supportive; ^6^ response from a 5-point scale ranging from no role to very helpful; ^7^ response from a 5-point scale ranging from strongly disagree to strongly agree; ^8^ when explained that the surgery would be covered by insurance with out-of-pocket costs up to $1000 and most patients are able to return to work in 3 weeks

## Results

### Characteristics of Participants

Among the study sample of 129 men, 71 (55%) were unwilling and 58 (45%) were willing to undergo MBS for obesity management (Table 1). The average age of the participants was 47 years (standard deviation, SD = 13), 49% were Black, mean BMI was 43 (SD = 5) kg/m^2^, 71% had some college or higher education (79% among MBS-unwilling vs. 63% among MBS-willing, *p* < 0.05), 79% were currently employed, 55% were currently married or living with a partner and 47% were ever smokers. Education level was the only participant characteristic different among the study groups, where 79% of men unwilling to undergo MBS completed some college or higher education vs 63% among those who were willing (p < 0.05).

Despite having a BMI of 35 or higher, 14% of the participants did not consider themselves to be suffering from obesity (Table 2). Men willing to consider MBS were less likely to be satisfied with their body size (27% vs. 48% among MBS-unwilling, *p* < 0.05) but were more likely to receive support from their physicians (55% vs. 32% among MBS-unwilling, *p* = 0.03) in their weight loss efforts and more likely to have physicians introduce them to the idea of metabolic and bariatric surgery (39% vs. 19% among MBS-unwilling). Men willing to consider MBS were more likely to know someone (community role models including friends or community leaders, whom the participants knew and had influence in their community) who underwent the surgery and had satisfactory results (66% vs. 40% among MBS-unwilling, *p* = 0.02). They also believed that MBS was a safe and an effective procedure (40% vs. 13% among MBS-unwilling, *p* < 0.01). More detailed results are presented in Supplementary Table 1.

### Multivariate Analysis to Examine Predictors of Considering MBS

**Fig. 1:**
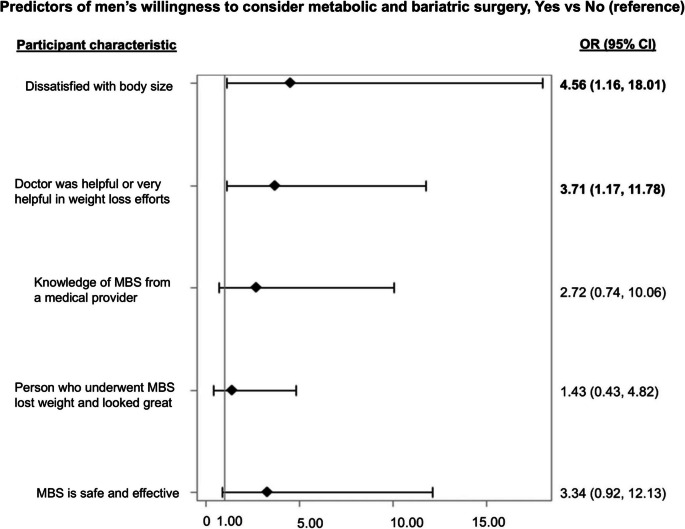
Forest plot showing results from multivariate analyses on participants’ consideration of metabolic and bariatric surgery based on perception of obesity, relationship with their physician and perception of safety and effectiveness of weight loss. Multivariable models adjusted for participants’ education level. Statistically significant results are represented in bold font. Abbreviations: MBS: metabolic and bariatric surgery, OR: odds ratio; CI: confidence intervals

Results from multivariable models to examine predictors of willingness to consider MBS after adjusting for education level (Fig. 1) showed that men who were dissatisfied with their body size (OR = 4.56, 95% CI: 1.16, 18.01) and those who had physicians helping them in their weight loss efforts (OR = 3.71, 95% CI: 1.17, 11.78) were more likely to consider surgery to lose excess weight. All other associations between men’s perceptions and willingness to consider MBS, including by race, were non-significant after adjusting for education level.

## Discussion

Overall, 45% of men with obesity in the study sample were willing to consider MBS, a much higher proportion than the 1% utilization of MBS among eligible individuals in the US [6]. The main factors associated with a positive attitude towards undergoing MBS among men included dissatisfaction about own body size and active physician involvement in their weight loss efforts. Men with more years of education were less likely to consider surgery for themselves and were more skeptical of the benefits of surgery, compared to men with fewer years of education. No racial differences in the attitude toward weight and weigh loss were detected in the study sample.

Body perceptions may play an important role in seeking care and making treatment decisions. In the current study, 86% of the men considered themselves to be suffering from obesity but only those who were dissatisfied with their body size (73%) were willing to consider MBS to manage their obesity. Men may be less likely to be dissatisfied with their body size than women because they are less likely to be subjected to societal ideals and pressures about their weight. This may dissuade them from considering MBS because of their perception that MBS is for cosmetic purposes only [24]. On the other hand, physicians’ beliefs that men may be less likely to be receptive to surgical treatment options unless it is a life threatening or disabling situation [24, 25] may lead to lower uptake of MBS among men with obesity. Wee et. al. (2013) reported that among eligible patients at primary care practices, men (14%) were less likely to be recommended for MBS compared to women (22%, *p* < 0.05) [9]. Another study that surveyed 820 U.S. bariatric surgeons found that among patients who met NIH criteria, gender didn’t affect their selection for surgery. However, for patients who didn't meet NIH criteria, men were 67% less likely to be selected for surgery [26]. This suggests that some surgeons may be influenced by social pressures on women to achieve body image ideals. In the current study, active physician communication and involvement in weight loss efforts was an important factor for men’s willingness to consider MBS. This emphasizes the physician’s role in shaping patient behavior and preferences [27, 28].

There is a large body of literature supporting the critical role of primary care providers in patients’ weight loss journey. PCPs have an important role in referring and providing short- and long-term postoperative care to achieve and maintain weight loss [29]. Earlier studies among PCPs revealed that physician hesitancy in referral to MBS is associated with lack of complete clarity on patient eligibility for surgical management as well as benefits and complications of MBS, ineffective communication with their patients on options for weight loss including safety and benefits of MBS, wanting to ‘do no harm’ lack of confidence in referring their patients for surgery, concerns about patient non-adherence to recommended dietary changes, and providing long-term postoperative care [30–33]. Therefore, both patients and PCP education is necessary to increase utilization of MBS among eligible men.

Clear and unambiguous communication between patients and physicians using culturally responsive messaging (i.e. communicating with respect and understanding of patients cultural background, beliefs and values [34]) and being empathetic and non-judgmental enables shared treatment decisions and should be a regular adopted practice for obesity management, including MBS [35–37]. Applying the principle of shared decision making summarized by Sarwer et al. (2021), if PCPs can initiate discussion on weight management with their patients considering their efforts until then, and whether MBS can alleviate more serious complications of obesity, it can also help with patient mistrust issues with medical community [38]. Further, communications with a multidisciplinary team that includes the PCP, surgeon, nursing, and rehabilitation staff can increase patient confidence and streamline care to achieve successful and sustained weight loss results [38].

In the current study, participants with some college or higher level of education were less likely to consider MBS to lose excess weight. Findings from earlier studies did not suggest the same. Wee et al., (2013) conducted telephone interviews among community clinic patients to understand patients’ willingness to undergo bariatric surgery, where education level was positively associated with patients’ willingness to consider MBS [9]. Another study by Stanford et al. (2015) among patients seeking bariatric surgery, those who had some college or higher education were more likely to undergo surgery within one year of the study interview [10]. Participants in the current study were different from the above studies as all of them were male and community residents who were not actively seeking bariatric surgery. Patients with higher education levels might feel more self-efficient to manage their weight by non-surgical means compared to less educated peers. Additionally, better educated participants might feel more confident in utilizing available tools and access to more options (for example gym memberships, personal trainers and being able to afford planned meals) to lose excess weight and might not perceive the need for surgical treatments. These findings should be tested and explored in further and larger studies in the future.

Several patient characteristics or socio-economic factors may influence the underutilization of MBS [8, 14, 39, 40]. Health insurance coverage and out of pocket costs are often significant barriers for patients to consider MBS for weight management [25, 41]. However, following enactment of Affordable Care Act (ACA) in 2010, the proportion of uninsured Americans decreased considerably from 18% prior to 2013 to 8% in 2023, and many insurance companies and Medicare, now cover bariatric surgery [42–45]. New York State provides more generous coverage for MBS than many other states [46, 47], and ranks among the top 5 states in access to MBS providers [48]. Yet, the rates of MBS in New York State are similar to the national level. Notably, lower utilization of MBS was also observed among socially-disadvantaged population in Canada, despite its public healthcare system [14].

Currently, most health plans under the ACA, including the public health insurance program for low income population (Medicaid), are required to cover annual nutrition counseling for people at high risk of chronic diseases, such as hypertension and type 2 diabetes, at no charge [49]. Such initiatives that include nutritional counseling provided by registered dietitians and other trained practitioners could improve access among those who are unable to afford these services. More research is needed to better understand factors affecting physician conduct or refer patients to nutrition counseling.

Despite providing important insights into care-seeking behaviors among men with obesity, this study has several limitations. Currently in New York state, 95% of the population have health insurance coverage [50]. Hence the results from this study may not be generalizable to other geographic locations, states with different healthcare delivery system and health insurance eligibility, ethnicities or men of younger age. Some of the current findings may also have been limited due to the smaller sample size and multiple testing and should be re-examined in larger population samples. Further studies are needed to identify and minimize systemic barriers to high quality medical and surgical care and better understand the impact of culturally tailored messaging about obesity and metabolic and bariatric surgery toward men. Men may need more role models and encouragement for to consider MBS for weight management. Best practices and lessons learned from gender-specific educational programs including addressing unique issues for men and targeting social media and community venues that are more of interest to men (51–53), may be informative in influencing social norms about obesity and weight loss.

## Conclusion

Self-perception of body size, physician support and awareness of successful surgical weight loss cases appear to be the important factors influencing utilization of surgical treatment for weight loss among men. More research is needed to understand how men prefer to receive practical advice about weight loss, timely referral to weight loss specialists and facilitate healthy and realistic body size perceptions through counseling and educational interventions.

## Supplementary Information

Below is the link to the electronic supplementary material.

Supplementary file1 (DOCX 39 KB)

## Data Availability

Data that support the findings in this study will be made available upon reasonable request.
