# Supplementary material for: Perceptions and Beliefs About Obesity and Bariatric and Metabolic Surgery Among Black and White Men
Source: Obes Surg. 2025 Apr 23;35(7):2510–7. doi: 10.1007/s11695-025-07878-6 (PMC12270968; doi:10.1007/s11695-025-07878-6)
Supplement: Supplementary file 1 — Supplementary file1 (DOCX 39 KB) [file 11695_2025_7878_MOESM1_ESM.docx]

**Supplementary Table 1.** Participants responses to survey questions on perceptions, opinions and beliefs on obesity and metabolic and bariatric surgery by their willingness to consider surgery to manage obesity and obesity complications.

| **#** | **Question/response** | **Total**  **n=129** | **Consider MBS** | | ***P^1^*** |
| --- | --- | --- | --- | --- | --- |
|  |  |  | **No**  **n=71**  **n (%)** | **Yes**  **n=58**  **n (%)** |  |
| **Perception of personal health and obesity** | | | | | |
| 1 | How would you rate your personal health over the past year? (Please select a response on the scale between poor (1) and excellent (5) health)? (n = 128)  1-3  4-5 | 84 (65.6)  44 (34.4) | 45 (64.3)  25 (35.7) | 39 (67.2)  19 (32.8) | 0.73 |
| 2 | Adults often have to juggle competing priorities such as work, family, friends, and self. On a scale of 1 through 10, how would you rank your personal health in order of importance in your life? (n = 127)  1-7  8-10 | 75 (59.1)  52 (40.9) | 39 (56.5)  30 (43.5) | 36 (62.1)  22 (37.9) | 0.53 |
| 6 | How important is maintaining a healthy body weight when it comes to your overall health? (Not important 1 2 3 4 5 very important) (n = 122)  1-2  3  4-5 | 10 (8.2)  30 (24.6)  82 (67.2) | 6 (9.0)  16 (23.9)  45 (67.1) | 4 (7.3)  14 (25.4)  37 (67.3) | 0.93 |
| 8 | How do you feel about your body size (Very dissatisfied 1 2 3 4 5 very satisfied)? (n=127)  1-2  3  4-5 | 78 (61.4)  34 (26.8)  15 (11.8) | 37 (52.1)  23 (32.4)  11 (15.5) | 41 (73.2)  11 (19.6)  4 (7.1) | **<0.05** |
| 9 | Do you think of yourself as overweight or obese? (n=127)  No  Yes | 18 (14.2)  109 (85.8) | 9 (12.9)  61 (87.1) | 9 (15.8)  48 (84.2) | 0.64 |
| 10 | Have other people told you that you are overweight or obese? (n=128)  No  Yes | 19 (14.8)  109 (85.2) | 10 (14.1)  61 (85.9) | 9 (15.8)  48 (84.2) | 0.78 |
| **Weight loss efforts** | | | | | |
| 11 | What factors do you think contributed in any way to your becoming overweight or obese?  Age  Diet  Lack of physical activity  Lack of sleep  Genetics  Stress | 94 (74.0)  123 (96.1)  118 (91.5)  97 (76.4)  102 (81.0)  101 (80.2) | 56 (78.9)  70 (98.6)  64 (90.1)  54 (77.1)  59 (83.1)  62 (87.3) | 38 (67.9)  53 (93.0)  54 (93.1)  43 (75.4)  43 (78.2)  39 (70.9) | 0.16  0.17  0.75  0.82  0.49  **0.02** |
| 12 | Have you tried to change your diet or exercise habits to lose weight?  Neither diet nor physical activity  Diet only or physical activity only  Diet and physical activity | 19 (14.8)  46 (35.9)  63 (49.3) | 14 (20.0)  20 (28.6)  36 (51.4) | 5 (8.6)  26 (44.8)  27 (46.6) | 0.07 |
| 13 | How supportive are your friends and/or family members in your efforts to lose weight?  Very supportive  Somewhat supportive  Not supportive/no role/make it harder | 67 (53.2)  35 (27.8)  24 (19.0) | 34 (48.6)  24 (34.3)  12 (17.1) | 33 (58.9)  11 (19.6)  12 (21.4) | 0.19 |
| **Relationship with physician** | | | | | |
| 3 | Where do you usually go to get routine medical care - when you are sick, medication refill or need advice about your health?  Single primary care provider (MD/NP/PA)  One or multiple providers for usual care of chronic disease  Single facility, seeing different providers.  Emergency room | 88 (69.9)  18 (14.3)  10 (7.9)  10 (7.9) | 45 (66.2)  14 (20.6)  3 (4.4)  6 (8.8) | 43 (74.1)  4 (6.9)  7 (12.1)  4 (6.9) | 0.07 |
|  | Among those with a single primary care provider. when discussing your weight, your doctor  Spoke respectfully (agree/strongly agree)  was judgmental (disagree/strongly agree) | 71 (83.5)  59 (70.2) | 35 (79.6)  30 (68.2) | 36 (87.8)  29 (72.5) | 0.57  0.94 |
| 4 | In the past year, how many times have you visited your usual or primary care provider?  Did not visit any provider.  1 time  2-5 times  6 times or more | 14 (10.8)  34 (26.4)  65 (50.4)  16 (12.4) | 9 (12.7)  19 (26.8)  32 (45.1)  11 (15.5) | 5 (8.6)  15 (25.6)  33 (56.9)  5 (8.6) | 0.46 |
| 14 | Has any healthcare provider ever talked to you about your weight?  No  Yes | 12 (9.7)  112 (90.3) | 6 (8.6)  64 (91.4) | 6 (11.1)  48 (88.9) | 0.64 |
| 15 | How has your doctor helped you with weight loss and/or maintaining a healthy weight?  No advice/plan  Advice only  Referrals/access to exercise equipment/linked to support group | 27 (16.0)  113 (67.3)  28 (16.7) | 17 (18.7)  59 (64.8)  15 (16.5) | 10 (13.0)  54 (70.1)  13 (16.9) | 0.60 |
| 16 | When discussing your weight, would you agree your doctor:  a. Spoke respectfully (Agree/strongly agree)  b. Appeared understanding of your situation (Agree/strongly agree)  c. Explained why maintaining a healthy weight is important for overall health (Agree/strongly agree)  d. Provided a detailed and realistic plan to help you lose weight (Agree/strongly agree)  e. Listened to your concerns and answered your questions (Agree/strongly agree).  f. Was judgmental (Disagree/strongly disagree).  g. Was not sincerely willing to help (Disagree/strongly disagree). | 100 (80.7)  96 (79.3)  104 (84.6)  58 (47.2)  85 (69.1)  83 (68.0)  83 (68.0) | 54 (77.1)  54 (78.3)  59 (84.3)  29 (41.4)  48 (70.6)  46 (66.7)  49 (71.0) | 46 (85.2)  42 (80.8)  45 (84.9)  29 (54.7)  37 (67.3)  37 (69.8)  34 (64.2) | 0.53  0.57  0.05  0.31  0.42  0.91  0.52 |
| 17 | In your opinion, what is the role of healthcare providers in addressing a patient's excess body weight? (You may check or select one or more responses)  a. Refer patients to other specialists.  b. Educate patients about the consequences of excess body weight.  c. Assist patients in setting goals for weight loss.  d. Hold patients accountable.  e. Recommend patients to metabolic and bariatric surgery.  f. Educate patients about healthy diet and exercise | 51 (15.0)  78 (22.9)  82 (24.1)  43 (12.6)  20 (5.9)  66 (19.4) | 25 (13.7)  48 (26.2)  44 (24.0)  20 (10.9)  7 (3.8)  39 (21.3) | 26 (16.6)  30 (19.1)  38 (24.2)  23 (14.7)  13 (8.3)  27 (17.2) | 0.23 |
| 18 | How helpful was your doctor in your efforts to lose weight (scale Not very helpful, 1 to Very helpful, 5) (n=127)  1-2  3  4-5 | 21 (16.5)  52 (40.9)  54 (42.5) | 13 (18.3)  35 (49.3)  23 (32.4) | 8 (14.3)  17 (30.4)  31 (55.4) | **0.03** |
| **Knowledge and attitude towards metabolic and bariatric surgery.** | | | | | |
| 19 | Have you ever heard about MBS?  No  Yes  From television/social media/print advertisements.  From a family member/co-worker.  From a medical provider. | 8 (6.3)  120 (93.7)  57 (47.5)  65 (54.2)  33 (27.5) | 3 (4.2)  68 (95.8)  37 (54.4)  32 (47.1)  13 (19.1) | 5 (8.8)  52 (91.2)  20 (38.5)  33 (63.5)  20 (38.5) | 0.47  0.08  0.07  **0.02** |
| 20 | Do you personally know anyone who underwent MBS?  No  Yes | 33 (26.2)  93 (73.8) | 15 (21.7)  54 (78.3) | 18 (31.6)  39 (68.4) | 0.21 |
| 20a | Do you think the quality of life and health improved for the person who underwent MBS after surgery? (n=93)  Strongly disagree/Disagree  Neither agree nor disagree  Agree/Strongly agree | 20 (21.5)  22 (23.7)  51 (54.8) | 8 (18.6)  14 (32.6)  21 (48.8) | 12 (24.0)  8 (16.0)  30 (60.0) | 0.17 |
| 20b | How do you rate the results of their MBS? (n=86)  Lost weight, feels and looks great.  Lost weight but feels and looks unhealthy/Did not lose weight. | 44 (51.2)  42 (43.0) | 19 (39.6)  29 (60.4) | 25 (65.8)  13 (34.2) | **0.02** |
| 21 | Do you think MBS is a safe and effective way to lose weight? (n=126)  Strongly disagree/Disagree  Neither agree nor disagree  Agree/Strongly Agree | 38 (30.2)  57 (45.2)  31 (24.6) | 29 (40.9)  33 (46.5)  9 (12.7) | 9 (16.4)  24 (43.6)  22 (40.0) | **<0.01** |

^1^ from Chi-square or Fisher’s Exact (where Chi tests may not have been valid)

Abbreviations: MBS: metabolic and bariatric surgery.
